# Metal phthalocyanines: thin-film formation, microstructure, and physical properties

**DOI:** 10.1039/d1ra03853b

**Published:** 2021-06-18

**Authors:** Rosemary R. Cranston, Benoît H. Lessard

**Affiliations:** University of Ottawa, Department of Chemical and Biological Engineering 161 Louis Pasteur Ottawa ON Canada benoit.lessard@uottawa.ca; University of Ottawa, School of Electrical Engineering and Computer Science 800 King Edward Ave. Ottawa ON Canada

## Abstract

Metal phthalocyanines (MPcs) are an abundant class of small molecules comprising of a highly conjugated cyclic structure with a central chelated metal ion. Due to their remarkable chemical, mechanical, and thermal stability MPcs have become popular for a multitude of applications since their discovery in 1907. The potential for peripheral and axial functionalization affords structural tailoring to create bespoke MPc complexes for various next generation applications. Specifically, thin-films of MPcs have found promising utility in medical and electronic applications where the need to understand the relationship between chemical structure and the resulting thin-film properties is an important ongoing field. This review aims to compile the fundamental principles of small molecule thin-film formation by physical vapour deposition and solution processing focusing on the nucleation and growth of crystallites, thermodynamic and kinetic considerations, and effects of deposition parameters on MPc thin-films. Additionally, the structure-property relationship of MPc thin-films is examined by film microstructure, morphology and physical properties. The topics discussed in this work will elucidate the foundations of MPc thin-films and emphasize the critical need for not only molecular design of new MPcs but the role of their processing in the formation of thin-films and how this ultimately governs the performance of the resulting application.

## Abstract 2

Metal phthalocyanines (MPcs) are an abundant class of conjugated small molecules comprising and their integration into thin films is critial for the proper function of next generation applications.

## Graphical abstract

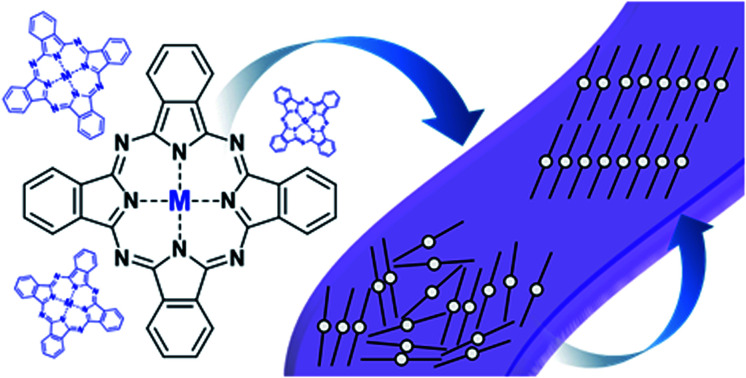

## 1. Introduction

**Fig. 1:**
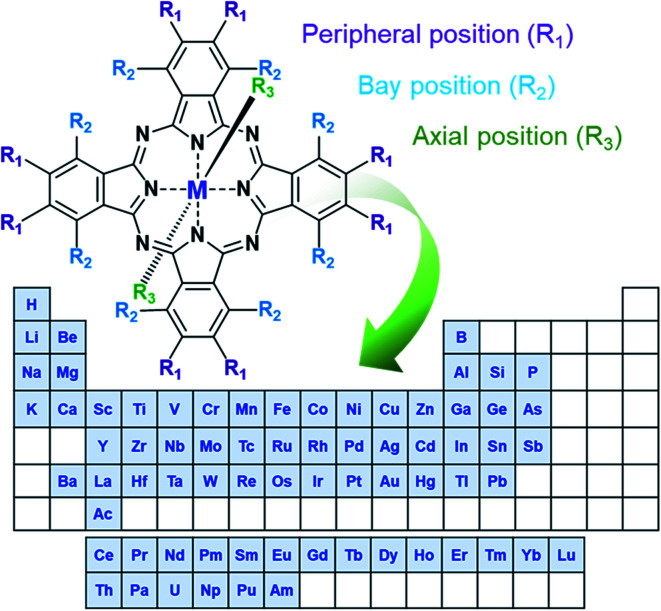
Schematic diagram of MPc structure with elements that form phthalocyanine complexes.^1,2^

In the simplest form, MPcs (C_32_H_18_N_8_M) consist of four isoindole groups connected by nitrogen atoms forming an 18 π-electron ring structure, with two covalent bonds and two coordination bonds chelating a metal or metalloid center (Fig. 1). With the possibility of over 70 central metal ions and 16 reactive sites in the peripheral and bay positions an astonishing number of MPc complexes are possible.^1,2^ Additionally, trivalent and tetravalent metal cations allow for the introduction of axial substituents providing an additional handle for tuning material properties. The choice of metal and the inclusion of peripheral, bay, or axial functionalization groups can strongly influence the physical and chemical properties of MPcs facilitating specific material tailoring. The extensive delocalization of the π-electron system and the exceptional stability of MPcs has resulted in their use for a myriad of applications since their discovery in 1907 and the first patent in 1929.^1,2^ Historically, due to their vibrant blue, purple, or green colour, MPcs have been, and are still, used as commercial colourants in paints, plastics, textiles, printing inks, dyes, and even some food colouring.^3^ Non-colourant applications have included catalysts, lubricants, indicators, and semiconductors, with recent interest focusing on more advanced applications.^3^ The ability of MPcs to form highly ordered thin-films coupled with their chemical and mechanical stability has led to their use as the active layer in a number of electrochemical and photo-electrochemical sensors for drug analysis and the detection of pharmaceutical products,^4^ gas sensing including the detection of alcohol vapours,^5–8^ cannabinoid sensing,^9^ and gamma radiation sensing.^10^ MPc thin-films are also a vastly growing area of research for emerging organic electronic devices having found promising success in organic photovoltaics,^11,12^ thin-film transistors,^13,14^ and light emitting diodes.^15^

In this review, we focus on the formation of MPc thin-films and their physical properties. The first section considers how thin-films of MPcs are formed from solid material by physical vapour deposition (PVD), highlighting the general principals of the nucleation and growth of organic small molecules, kinetic and thermodynamic considerations, and effects of deposition parameters. The second section focuses on MPc thin-films formed from solution, with a discussion on the relevant nucleation principles and a comparison of solution deposition methods. The third section illustrates the general microstructure of MPc thin-films with an examination of the commonly seen packing motifs, polymorphs, and film morphologies. The fourth section focuses on specific physical properties of MPc thin-films, mainly the optical absorption and vibrational properties which are most relevant to emerging photophysical MPc applications. Lastly, the final section reviews some of the most relevant and promising synchrotron based X-ray techniques which can be used to characterize and study MPc thin-films.

## 2. Thin-film growth of organic small molecules by physical vapour deposition

### 2.1 Physical vapour deposition

**Fig. 2:**
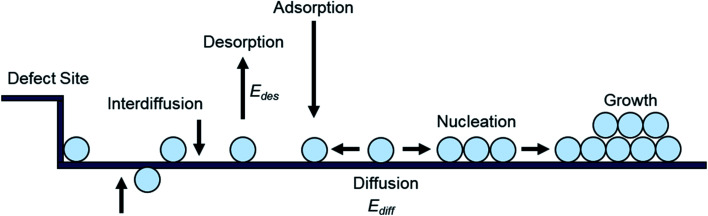
Schematic diagram of nucleation and growth processes on a substrate.

Small molecule thin-films are commonly fabricated by PVD, where under high vacuum (10^−6^ to 10^−8^ torr) the solid deposit material is heated above its sublimation temperature creating a vapour which then condenses on a target substrate. Numerous PVD techniques exist that employ different heating sources/mechanisms or different processing conditions but in all cases no vapour phase chemical reaction occurs such that thin-films are produced strictly through physical means. As the vapour reaches the substrate, thin-film formation proceeds through the nucleation and growth of molecules of the deposited material.^16–19^ While on the substrate, the free energy of the deposited molecules is reduced from that of the vapour phase, creating a low-density distribution, randomly diffusing among surface lattice sites.^16–19^ Molecules in this distribution may then diffuse across the substrate until they are lost by one of several processes (Fig. 2). The molecules may re-evaporate back into the vapour phase (desorption), nucleate to form 2D or 3D growth, aggregate into existing nucleation clusters, get captured at defect sites, or diffuse into the substrate (interdiffusion).^18–21^ For perfectly flat surfaces molecule capture at defect sites and interdiffusion are excluded from these possibilities, however in practice due to imperfections on the substrate these process often occur.^16,20,21^ After the initial formation of nucleation clusters, rearrangement to more thermodynamically stable forms can also occur. This can include mixing of different species, and shape changes caused by surface diffusion or coalescence brought on by post deposition treatments such as annealing. Thus, diffusion processes occur at several stages of thin-film formation, including the formation, mobility, and rearrangement of nucleation clusters.^16,19–21^

### 2.2 Thermodynamics and kinetics

Nucleation occurs in the beginning stages of phase change when a new phase forms from a prior parent phase often as a result from a change in temperature that triggers vapour-phase condensation, solidification, or solid-state phase transformations.^17–19^ In thin-film formation the initial nucleation stage often dictates the resulting grain structure, film morphology, and thin-film properties. The principal theories of inorganic thin-film growth can largely be used to model the nucleation behaviour of organic small molecules, however some fundamental differences do exist. Most notably, inorganic atoms are assumed to be isotropic in shape such that the orientation of the atom relative to the substrate is irrelevant, whereas many organic small molecules are highly anisotropic and thus the strength of the molecule–molecule and molecule–substrate interaction will depend on their orientation to the substrate.^20,22,23^ Additionally, inorganic film growth relies on strong covalent or ionic bonds, whereas organic materials rely on van der Waals interactions.^22,23^

For the vapour deposition of thin-films the thermodynamic driving force for nucleation is the difference between the chemical potential of organic molecules in the vapour phase (*μ*_v_) and crystalline phase (*μ*_c_).^16,17,20,24^ The Gibbs free energy change (Δ*G*) needed to form a finite-sized crystal composed of a number, *j*, of molecules can be described by:1
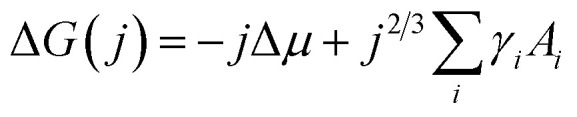
where the first term (−*j*Δ*μ*) describes the thermodynamic driving force, defined as the difference in chemical potentials Δ*μ* = *μ*_c_ − *μ*_v_, and the second term 
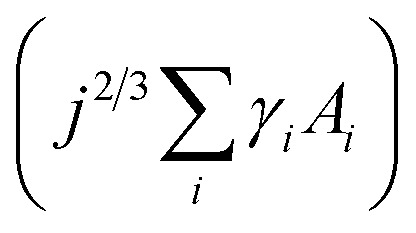
 describes the energy associated with creating or adding to a new surface.^16,17,20,24^ The term *γ*_*i*_ corresponds to the surface energy associated with surface *i* with an area *A*_*i*_.^16,17,20,24^Eqn (1) gives the macroscopic relationship in terms of free energy, between crystal size and surface energies and is a reasonable approximation of nucleation behaviour. In general, the barrier to nucleation where the surface energy effects are greatest (ΔG*) can be determined by setting the derivation of eqn (1) with respect to the number of molecules (*j*) to zero, this represents the point at which thermodynamic equilibrium is achieved. However, due to the anisotropic nature of organic molecules, nucleation is often governed by kinetic processes rather than thermodynamic ones.^20,22,23^ Therefore, thin-film growth is better described as a non-equilibrium kinetic process resulting in a macroscopic state that is dependent on the respective rates of the different physical processes illustrated in Fig. 2.^20,21^

Atomistic theories of nucleation describe the role of individual atoms, or molecules, during the initial stages of thin-film formation.^17,18,20,21^ An important advantage of the atomistic models is that nucleation can be expressed in terms of measurable parameters such as deposition rate and substrate temperature, instead of quantities such as ΔG* and *γ*_*i*_, whose values cannot be known with certainty or easily estimated.^17,20,21^ By this approach, the most simplified kinetic rate equation relating the time dependent change in monomer cluster density to surface processes is given by the following:2
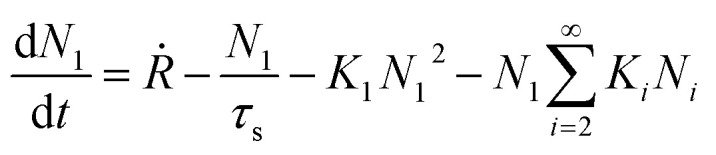
where *N*_1_ is the monomer density, *Ṙ* is the deposition rate, *τ*_s_ is the length of time atoms remain on the substrate before desorption, *N*_*i*_ is the critical concentration of clusters per unit area of size *i*, and *K*_1_ is a second-order rate constant.^17^Eqn (2) states that the monomer density change with time is given by the deposition rate, minus the desorption rate, minus the rate at which two monomers combine to form a dimer, minus the loss in monomer population due to their capture by larger clusters.^17,18,20,21^ This equation can be generalized further to define the rate equation for clusters of *i* size:3
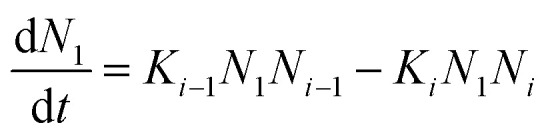
where the first term expresses the increase in rate caused by the attachment of monomers to smaller *i* − 1 sized clusters, and the second term describes the decrease in rate due to formation of larger *i* + 1 sized clusters.^17,19^ While eqn (2) and (3) are valuable in understanding the basic kinetics of nucleation, the inclusion of surface diffusion terms, coalescence, and transient and steady-state solutions offer a much more complete account of nucleation events, however increases the mathematical and physical complexity of these models greatly. More rigorous kinetic models can be found in other works.^18,20,21,23,25–27^

### 2.3 Nucleation density

For vapour deposited materials the rate of heterogeneous nucleation, defined as the number of stable clusters that form per unit volume per unit time, is a function of the deposition rate, substrate temperature, substrate surface properties, intermolecular interactions, and molecule–surface interactions.^16,17,20,24^ Greater nucleation rates typically result in fined grained thin-film morphologies due to the large number of crystallites that nucleate on the substrate in a short period of time.^16,17^ Conversely, if the nucleation rate is low large crystal growth is favoured.^16,17^ In terms of the energetic contributions, the energetic barrier to diffusion (*E*_diff_), energetic barrier to desorption (*E*_des_), and thermodynamic barrier (ΔG*) are critical to heterogeneous nucleation and thin-film growth.^16,17,20,24^ Considering these energetic terms, the nucleation density (*N*_D_) of stable clusters is given by eqn (4):4
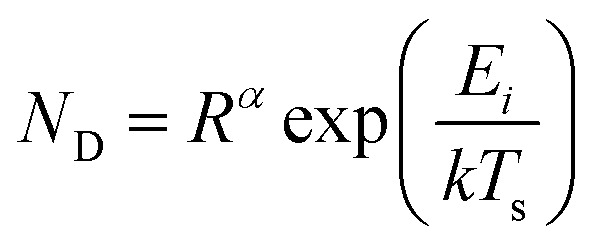
where *α* is a constant related to the critical cluster size, *k* is Boltzmann's constant, *T*_s_ is the substrate temperature, and *E*_*i*_ is the crystal disintegration energy defined as the energy required to disintegrate a critical cluster containing *i* molecules into *i* separate molecules.^16,17,20,24^ For systems with a low crystallization driving force, *E*_*i*_ is approximately equal to negative the crystal formation energy which can be approximated by *E*_*i*_ = (−*E*_des_ + *E*_diff_ + ΔG*) for the vapour deposition of most organic small molecules.^16,17,20,24^ Thus the three energetic barriers (diffusion barrier, desorption barrier and thermodynamic barrier) directly impact the nucleation density. The relationship between the energetic terms of eqn (4) and surface interactions of the substrate show that the processes illustrated in Fig. 2 (diffusion, desorption, and nucleation) are therefore a function of the interaction between the substrate and deposit material.^16,17,24^

### 2.4 Growth modes

**Fig. 3:**
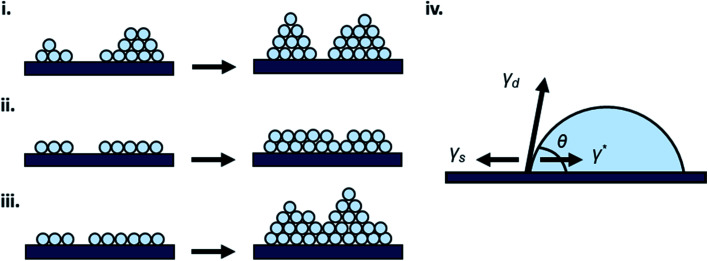
Diagram of (i) island, (ii) layer-by-layer, (iii) SK thin-film growth, and (iv) relevant surface energies.

Thin-film formation is generally characterized by three basic growth modes: island (Volmer–Weber), layer-by-layer (Frank–Vander Merwe), and Stranski–Krastanov (SK) growth. Island growth occurs when molecules of the deposited material are more strongly attracted to each other than to the substrate, resulting in 3D growth (Fig. 3i).^16–18,20,24^ Layer-by-layer growth exhibits the opposite characteristics as the molecules are more strongly attracted to the substrate resulting in planar 2D sheet formation often referred to as epitaxial growth (Fig. 3ii).^16–18,20,24^ SK growth describes the formation of one or more complete monolayers where subsequent 2D growth is unfavourable and 3D island growth continues (Fig. 3iii).^16–18,20,24^ Typically, organic thin-films, such as those composed of MPcs, experience SK growth.

The relationship between growth mode, surface energy of the deposited material, and the substrate can be related by eqn (5):

5 *γ*_s_ = γ* + *γ*_d_ cos *θ*

where *γ*_s_ is the surface energy of the substrate, γ* is the interfacial surface energy between the deposited material and substrate, *γ*_d_ is the surface energy of the deposited material, and *θ* is the contact angle (Fig. 3iv).^16–18,20^ In the case of layer-by-layer growth the deposited material wets the substrate and *θ* ≈ 0, therefore, *γ*_s_ ≥ γ* + *γ*_d_.^16–18,20^ However, for island growth the opposite is true and *θ* > 0, therefore, *γ*_s_ < γ* + *γ*_d_.^16–18,20^ SK growth combines features of both island and layer-by-layer growth where initially *γ*_s_ > γ* + *γ*_d_ until island formation occurs.^16–18,20^

### 2.5 Effect of deposition parameters

**Fig. 4:**
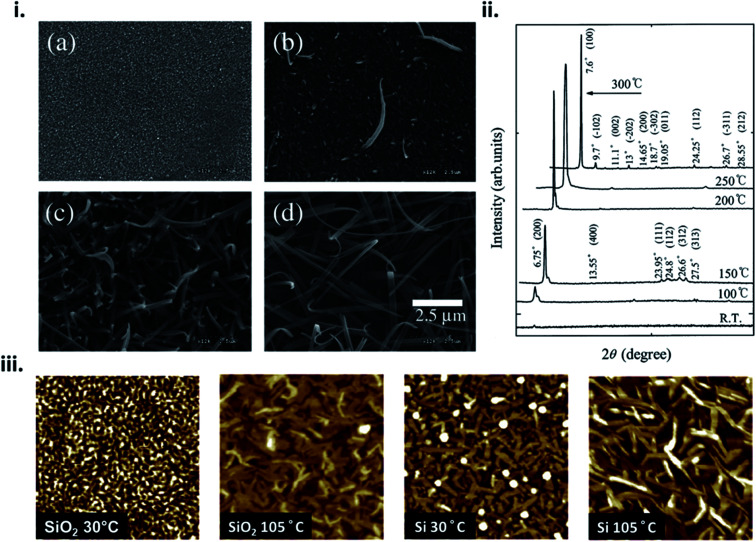
(i) SEM micrographs of CuPc thin-films deposited at (a) room temperature, (b) 150 °C, (c) 200 °C, and (d) 300 °C and (ii) XRD spectra of CuPc thin-films deposited at various temperatures. Adapted with permission from ref. 30. Copyright© 2002 Elsevier Science B. V. (iii) AFM images (1 μm × 1 μm), of CuPc thin-films deposited on SiO_2_ and Si substrates at substrate temperatures of 30 °C and 105 °C. Adapted with permission from ref. 28. Copyright© 2013 Elsevier B. V.

The formation of thin-films by PVD is a complex process that can be influenced by many factors such as material properties, deposition parameters, and environmental constraints resulting in film microstructure ranging from the formation of single crystal, polycrystalline, to amorphous films. From eqn (4) nucleation density is largely reliant on substrate temperature and deposition rate. Due to the Arrhenius nature of eqn (4), at elevated substrate temperatures the overall barrier to heterogeneous nucleation is reduced.^16,17,20,24^ At high substrate temperatures, molecules have increased kinetic energy and are able to easily migrate to lower energy sites creating nucleation points, resulting in polycrystalline structures with large crystallites and fewer grain boundaries.^24,28,29^ This phenomenon has been well documented in MPcs^28–36^ which at room temperature exhibit fine grained morphologies, whereas large rod-like fibers occur at increasing substrate temperatures, as exhibited by scanning electron microscopy (SEM) and atomic force microscopy (AFM) images of copper phthalocyanine (CuPc) in Fig. 4.^28,30^

For fabrication of MPc thin-films by PVD, substrate temperatures between 30–120 °C are commonly used leading to morphologies with large regular crystals and minimal grain separation, which tends to be preferable for various applications.^28,31–33^ At greater substrate temperatures (>200 °C) the sticking coefficient of the deposited material is reduced and nucleation is limited, resulting in a sparse network of very large crystallites separated by wide gaps (Fig. 4i).^30,31,34–36^ At very low temperatures (<0 °C) the surface mobility and diffusion are decreased such that molecules lack the energy required to find favourable nucleation cites, and amorphous films are formed, as illustrated by low temperature depositions of pentacene.^23,37,38^

**Fig. 5:**
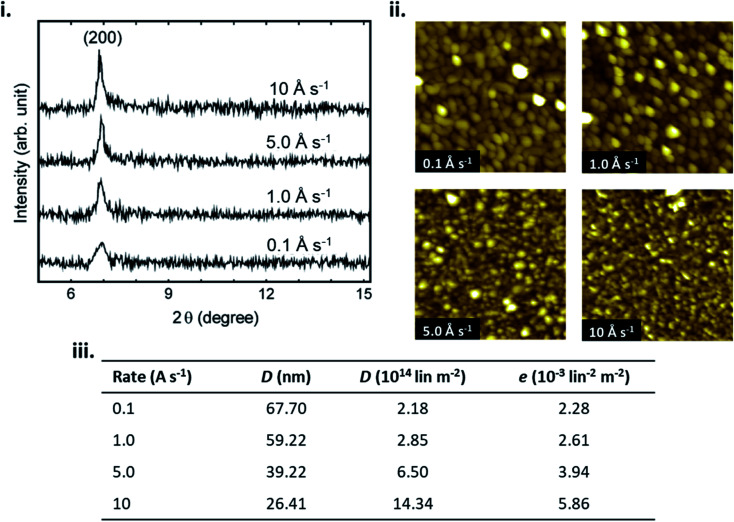
(i) XRD pattern of CuPc thin-films, (ii) AFM images (1 μm × 1 μm) of CuPc thin-films, and (iii) crystal size (*D*), dislocation density (*d*) and lattice microstrain (*e*) of CuPc thin-films, deposited at various deposition rates. Adapted with permission from ref. 40. Copyright © 2012 Elsevier Ltd.

In addition to substrate temperature, deposition rate effects the nucleation density and subsequently thin-film formation by determining the density of molecules diffusing on the surface. Increasing the deposition rate increases the rate of nucleation, as more molecules can interact to form a stable cluster in a defined area per unit time, often leading to smaller and denser crystallites.^23,39–41^ Conversely, decreasing the deposition rate decreases nucleation density as this allows more time for incoming molecules to migrate to a favourable orientation prior to the arrival of additional molecules.^23,39–41^ Low deposition rates typically lead to large crystallites, and fewer grain boundaries.^28,29,39,40^ Similar to substrate temperature, the effects of deposition rate on the fabrication of a variety of MPc thin-films has been extensively studied^28,39–41^ with Fig. 5 displaying the effects on CuPc films.^40^ If the deposition rate is very high, growth becomes kinetically dominated and typically low crystallinity, polycrystalline, or amorphous film formation is observed.^28^ For the fabrication of MPc thin-films deposition rates of 0.01–5 Å s^−1^ are commonly used as they generally result in morphologies with large crystallites, favourable π–π stacking, connected grain boundaries, and greater crystallinity, which are typically desired for many solid state applications.^28,39–41^

**Fig. 6:**
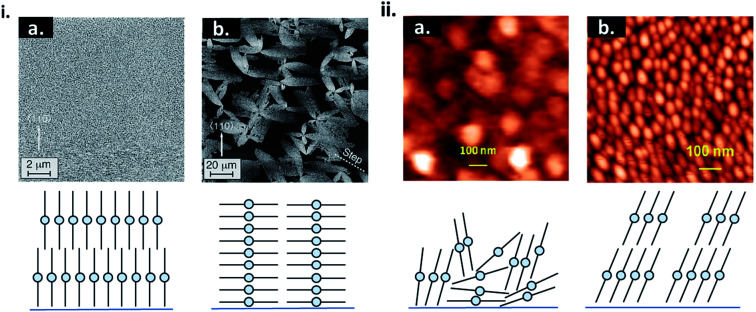
(i) AFM images CuPc thin-films and cross sectional diagrams CuPc molecules deposited on (a) rough and (b) smooth Si(111)–H surfaces. Adapted with permission from ref. 43. Copyright© 1996 American Vacuum Society. (ii) AFM images of CuPc thin-films and diagrams of CuPc molecules deposited on (a) bare SiO_2_, and (b) OTS treated SiO_2_. Adapted with permission from ref. 49. Copyright© 2015 American Chemical Society.

Physical surface roughness and surface chemistry of the substrate can have a significant impact on nucleation and thin-film formation. Areas of high surface roughness decrease the barrier for heterogeneous nucleation by decreasing the diffusion distance of molecules.^16,42–47^ This results in small grain formation, enhanced defects, and often a different molecular orientation relative to the substrate as exhibited by thin-films of CuPc (Fig. 6i).^42–44^ Altering the surface chemistry of the substrate through self-assembled monolayers (SAMs) is a common strategy to influence the morphology and crystallinity of small molecule thin-films. SAMs are highly ordered 2D structures consisting of a head group, terminal group, and linker. The head group typically has a specific affinity for a substrate which facilitates spontaneous monolayer formation.^48^ The most common SAMs used in thin-film engineering are thiols on gold, silanes on silicon oxide (SiO_2_), and phosphonic acids on metal oxides.^48^ Using SAMs to modify the substrate can affect the uniformity, morphology, packing structure, and molecular orientation of the resulting thin-film, as seen by the growth of CuPc on bare SiO_2_*versus* SiO_2_ treated with trichloro (octyl)silane (OTS) (Fig. 6ii).^45,48,49^ As discussed, the surface energy of the substrate will greatly influence the initial nucleation behaviour of the deposited material and determine the final growth mode.^17,20,45,48^ By using SAMs to selectively tune surface energy, island, layer-by-layer, and SK growth can be achieved using the same deposited material and fabrication conditions.^50,51^ Overall PVD is an effective fabrication method, already employed in industry and used for the engineering of thin-films with tunable molecular structures.

## 3. Thin-film growth of organic small molecules by solution processing

### 3.1 Solution deposition

**Fig. 7:**
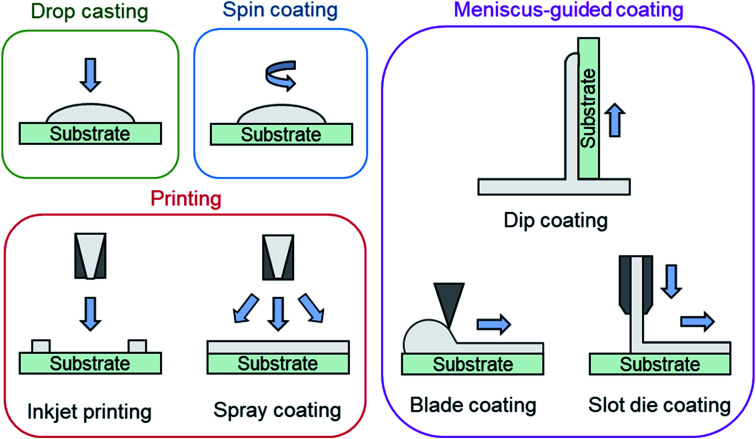
Diagram of solution processing methods mentioned in text.

Solution deposition of organic small molecules involves the dissolution of the deposit material into an organic solvent where it can then be deposited onto a substrate by one of four main methods: drop casting (also referred to as dispensing), spin coating, printing, and meniscus-guided coating (Fig. 7). As the solvent evaporates the solution becomes supersaturated, driving nucleation and crystal growth, to form a thin-film. Compared to PVD, the nucleation and growth of solution deposited materials is more complex due to added solvent–vapour, solvent–substrate, solute–solvent, and solute–substrate interactions.^52^ Additionally, control over the formation of thin-films by solution processes is limited due to the rapid progression of nucleation, crystallization, and growth stages that can occur in a matter of seconds.^53^

Drop casting and spin coating are common lab scale techniques used to deposit material on small area substrates. Drop casting involves depositing solution droplets onto a stationary substrate with controlled droplet size and momentum, where the solvent is left to slowly evaporate, leading to the formation of a thin-film.^52^ As no outside forces are applied, nucleation begins along the edge of the droplet with crystal growth occurring in the direction of the contact line recession. Drop casting can often lead to non-uniform deposition since the recession of the contact line is typically irregular. Spin coating is a more consistent fabrication method used to create uniform thin-films by dropping solution onto a rotating substrate which simultaneously spreads the solution by rotational forces while quickly evaporating the solvent.^52^

Printing is a broad definition of different deposition techniques, however it typically refers to large area solution processing methods that do not primarily rely on directional shear-induced alignment such as meniscus-guided coating.^52^ Inkjet printing is one of the most common and popular printing methods where a jet of solution is ejected from a chamber by a piezoelectric or thermal actuator and is deposited onto a substrate.^52^ Similar to inkjet printing, spray coating ejects solution from a nozzle where small droplets are formed by aerosolization with an inert gas and are deposited onto the substrate.^52^ Inkjet printing and spray coating are particularly useful as their compatibility with roll-to-roll manufacturing facilitates effective high throughput fabrication.

Meniscus-guided coating methods are scalable large area techniques that use the linear movement of either the substrate, or coating tool, to fabricate thin-films with uniformly aligned crystal growth.^52,54^ Dip coating, involving the vertical withdrawal of a substrate from a solution bath, blade coating, involving the use of a flat rectangular edge to spread solution across a substrate, and slot die coating, involving the flow of solution through an orifice and shaping device onto a horizontally moving substrate are common examples of meniscus-guided coating methods.^52,54^ The alignment and size of the growing crystallites relies on the shear force directing solution flow and is largely influenced by the speed at which movement occurs.

### 3.2 Thermodynamics and kinetics

When a solution is introduced onto a substrate surface, solvent evaporates, increasing the concentration of the solution until it becomes supersaturated and the dissolved molecules begin to precipitates to form a thin-film. The formation of precisely controlled thin-films with specific grain structures and morphologies remains a challenge for solution processing due to the rapid nucleation and growth steps. The same thermodynamic principals that describe PVD apply to solution deposition such that the thermodynamic driving force for nucleation is the difference between the chemical potential of organic molecules in the liquid phase (*μ*_l_) and crystalline phase (*μ*_c_).^55^ In the case of solution deposition, Δ*μ* corresponds to the difference between the concentration of the solution at equilibrium (*C*_∞_) and the concentration during growth (*C*), which can be expressed as a function of the substrate temperature (*T*_s_):^55^6
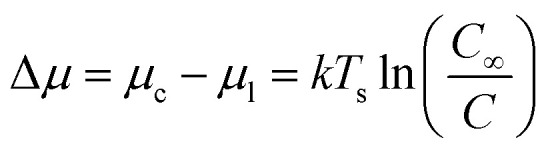


Thermodynamically, *C* and *T*_s_ are the two thermodynamic parameters that determine the nucleation and growth of crystallites during solution deposition, however, similar to PVD, solution deposition methods are largely governed by kinetic processes and rates of crystallization.^56^ In the case of solution deposition, the kinetic driving force for nucleation is the rate of solvent evaporation which directly determines the rate of crystallization, and is thus key to the fabrication of consistent small molecule thin-films.^52,57^ Due to variations in the respective solution processing methods the governing principals for the rate of solvent evaporation will be method specific.

Drop casting and printing techniques use the release, impact, and spreading of one or more solution droplets that may form a continuous thin-film before drying or may dry individually to create a thin-film composed of many islands. Controlling the rate of solvent evaporation, and thus the nucleation and growth stages, depends solely on the solvent and substrate properties as no external rotational or shear forces are applied.^58–60^ The solution and substrate surface properties can influence the deposition by causing splashing, spreading, receding, and/or rebounding.^61–63^ Additionally, temperature and concentration gradients within solution droplets can lead to coffee ring and Marangoni effects, leading to poorly controlled film formation.^59,60^

Thin-film formation by spin coating can be accurately represented when the evaporation rate of the solvent, the viscosity increase resulting from the increase in solute concentration, the surrounding vapour phase above the substrate, and the solvent's properties are taken into account.^64,65^ The simplest and earliest models describing liquid flow on a rotating surface are formulated with three main assumptions: (i) the gas and liquid phases are Newtonian fluids; (ii) the fluid flow is axially symmetric and laminar; and (iii) the rotating surface is flat and extends infinitely.^64,65^ It is widely accepted that the early stages of spin coating are flow dominated while late stages are dominated by the rate of solvent evaporation. At the transition point, when evaporation and flow become equal, the evaporation rate (*ν*_e_) depends on the rotational speed (*ω*), yielding:^64–68^

7 *ν*_e_ = *ω*^1/2^

This simple relationship has been observed experimentally using polymer thin-films with only small reported variations in the exponent value.^64,65,68–72^ However, as solvent evaporates the physical properties of the solution change, inducing non-Newtonian behavior. More rigorous models describing the spin coating process take into account heat and momentum transfer by including the effects of solution viscosity and solvent volatility.^65,70,71^ The two stage flow dominated and evaporation dominated process of spin coating has been corroborated with experimental data from spin coated small molecule thin-films by *in situ* grazing-incidence wide-angle X-ray scattering (GIWAXS).^73,74^ These experiments show how the rapid flow dominated crystallization stage, which occurs over a sub-second time scale, is independent of the rotational speed, and the slower more gradual evaporation dominated stage is rotation speed dependant.^73,74^ Therefore, the rate of solvent evaporation during spin coating can be described by eqn (7).

Meniscus-guided coating methods depend mainly on solvent properties and coating speed. Solvent evaporation is dominate in the meniscus region leading to supersaturation, precipitation, and ultimately to nucleation. However, most meniscus-guided methods use an external shear force to enhance thin-film uniformity and crystallite alignment. The intensity of this force, determined by the coating speed (*ν*_c_), can be divided into two categories: fast coating speeds (*ν*_c_ ≈ 1 mm s^−1^) and slow coating speeds (*ν*_c_ ≈ 1–100 μm s^−1^). Fast coating speeds where solution is spread out by shear forces and solvent evaporation is separated from the meniscus region is known as the Landau–Levich–Derjaguin (LLD) deposition regime where solvent evaporation is a function of *ν*_c_.^75–77^ At slow coating speeds deposition corresponds to the evaporation regime where *ν*_c_ is approximately equal to *ν*_e_ of a pinned drop of solution that is receding primarily due to evaporative mass loss.^77^ Thus, in contrast to the LLD regime where solvent evaporation is separate from thin-film deposition, the evaporation regime is complicated by the interactions between solvent evaporation, fluid flow, and film formation.^75–77^ A number of models have been purposed to describe *ν*_e_ most of which take on the general form of eqn (8).^54,77,78^8
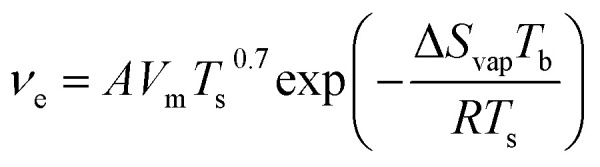
Here *V*_m_ is the molar volume of the liquid solvent, Δ*S*_vap_ is the entropy of vapourization of the solvent, *T*_b_ is the boiling point of the solvent at atmospheric pressure, and *A* is a single fitting parameter combining all temperature independent variables.^77,78^

### 3.3 Effect of deposition parameters

**Fig. 8:**
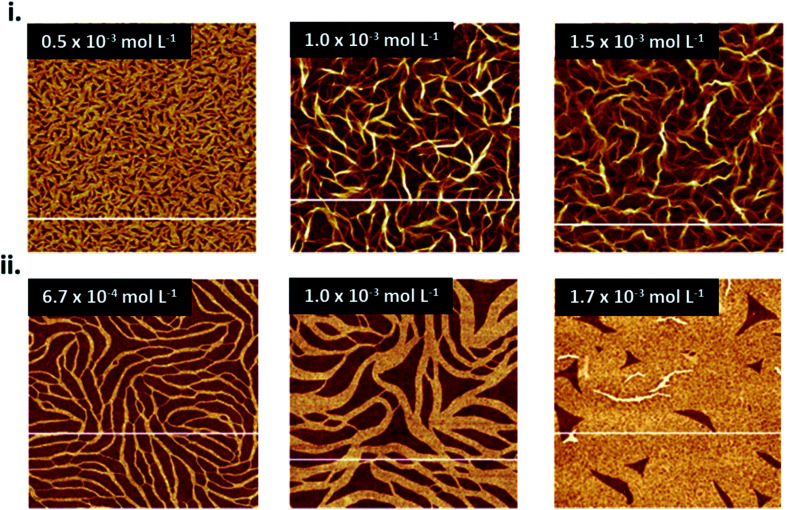
AFM images (5 μm × 5 μm) of CuPc thin-films fabricated by (i) spin coating and (ii) dip coating at various concentrations of CuPc solution on SiO_2_ substrates. (i) Adapted with permission from ref. 79. Copyright© 2020 the Authors under the Creative Commons Attribution Non-Commercial No Derivatives 4.0 License. (ii) Adapted with permission from ref. 80. Copyright© 2015 Elsevier B. V.

Solution deposition processes can produce wide variations in thin-film microstructure depending on solution concentration, solvent type, substrate temperature, and substrate surface chemistry. Solution concentration influences thin-film coverage, such that at low concentrations low coverage sub-monolayer formation is observed, whereas at increasing concentrations the coverage and interconnectivity increase with the formation of mesh layers and multilayers. This phenomena has been documented in spin coated and dip coated CuPc thin-films where, at low solution concentration, CuPc molecules form a sub-monolayer of interconnected ribbons typically 20–50 nm wide, approximately 100 nm in length, and 1 nm thick (Fig. 8).^79–81^ As the concentration of CuPc in the deposited solution increases, multiplayer formation is observed, however complete coverage for a single layer is never achieved due to the anisotropic nature of CuPc which effects surface diffusion and subsequent nucleation.^79–81^

Solvent choice plays an important role in the formation of thin-films by solution deposition. As discussed, the rate of solvent evaporation directly determines the crystallization rate, dictating the final thin-film morphology and microstructure. Solvents with a faster rate of evaporation generally leads to films with a greater surface roughness due to the occurrence of well separated clusters. Solvents with high evaporation rates, such as chloroform, can lead to the formation of these clusters since the rapidly evaporating solvent leaves little time for surface mobility or diffusion of the molecules on the substrate. This often results in lower aggregation and films with a non-coalesced morphology. Solvents with low evaporation rates, such as dimethylformamide, facilitate greater molecular mobility on the surface due to the longer evaporation time and often results in a more highly packed and ordered film. This has been demonstrate with tetrakis-(isopropoxy-carbonyl)-copper phthalocyanine (TIP-CuPc)^82^ and a number of other semiconducting small molecules.^58,83–85^

**Fig. 9:**
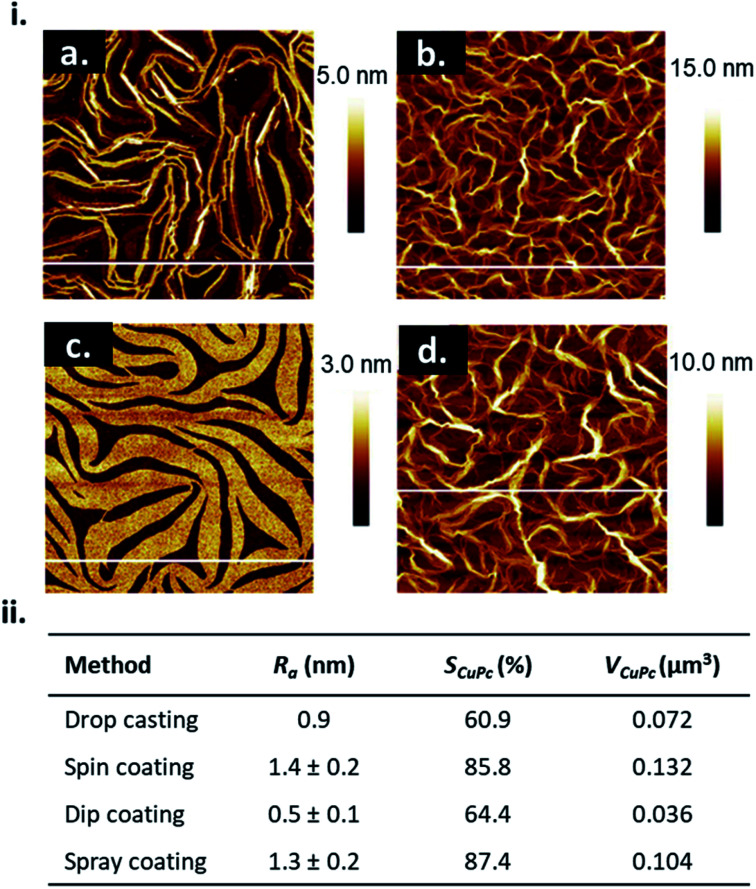
(i) AFM images (5 μm × 5 μm) of CuPc thin-films fabricated by (a) drop casting, (b) spin coating, (c) dip coating, and (d) spray coating. (ii) Mean roughness (*R*_a_), substrate coverage fraction (*S*_CuPc_), and film volume (*V*_CuPc_) for CuPc films deposited by solution processing methods. All films were fabricated from 1.5 × 10^−3^ mol L^−1^ CuPc solution on SiO_2_ substrates. Adapted with permission from ref. 79. Copyright© 2020 the Authors under the Creative Commons Attribution Non-Commercial No Derivatives 4.0 License.

The choice of solution processing method will have significant influence on thin-film microstructure. A recent study by Gojzewski *et al.*, exhibited the differences in CuPc film formation by drop casting, spin coating, dip coating, and spray coating (Fig. 9).^79^ The authors used CuPc dissolved in trifluoroacetic acid (TFA) that immediately spreads to cover the hydrophilic surface of SiO_2_ to form a liquid film. Upon drop casting, outward capillary flow from the center of the drop brings dissolved CuPc molecules to the edge, creating the morphology shown in Fig. 9i, a. Spin coating using the same solution produced a multi-layer formation of nanoribbons similar to that of drop casting (Fig. 9i, b), however the added rotational force increases the rate of solvent evaporation creating a rougher film surface (Fig. 9ii).^79^ Dip coating yielded similar film characteristics (roughness, coverage and film volume) to drop casted films, however exhibited a unique morphology consisting of a sub-monolayer mesh-like film made of long, asymmetrically curved and interconnected nanoribbons approximately 600 nm wide where the CuPc molecules were orientated in-plane to the substrate (Fig. 9i, c).^79^ Spray coated films displayed a similar morphology and comparable surface roughness, coverage, and film volume to spin coated films with large rod-like CuPc aggregates (Fig. 9i, d).^79^ Due to the added rotational force during spin coating, noticeable differences in film morphology between the two fabrication methods are expected. However, as discussed, morphology is dependent on the rate of solvent evaporation. The specific fabrication parameters used for spray coating and spin coating in this case allows for sufficient TFA evaporation to create films of large rod-like CuPc aggregates.^79^ This further corroborates the relationship between thin-film microstructure and solvent evaporation as the driving force for the nucleation and growth of solution deposited thin-films.

## 4. Thin-film microstructure of metal phthalocyanines

### 4.1 Packing motifs

**Fig. 10:**
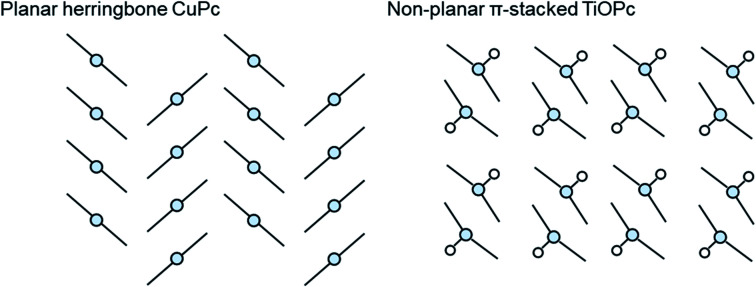
Schematic diagram of herringbone crystal packing represented by CuPc and π-stacked crystal packing represented by TiOPc.

The growth mode and packing structure of inorganic thin-films is well understood by reason of the strong covalent bonds, and the inherent isotropic shape of inorganic atoms. In contrast, due to the anisotropic geometry and weak van der Waals forces of organic molecules more variable crystallite growth, molecular packing structures, thin-film textures, and morphologies are observed.^86,87^ Molecular packing can not only impact the solid state properties of organic molecules but it can also effect the thermodynamic, kinetic, mechanical, electrical, and optical properties of the final thin-film.^88^ The identification and classification of different packing structures is therefore crucial for applications in various industries including pharmaceuticals,^89^ organic semiconductors,^90^ pigments,^91^ and explosives.^92^ Conjugated aromatic small molecules have been known to form two main crystal packing structures: herringbone and π-stacked (Fig. 10).^93^ The herringbone structure exhibits altering face-to-edge and face-to-face molecular packing, and mainly occurs in planar MPcs such as CuPc, silicon phthalocyanine (SiPc) and zinc phthalocyanine (ZnPc), whereas the π-stacked configuration exhibits face-to-face packing and is adopted by non-planar MPcs such as titanyl phthalocyanine (TiOPc), chloro-aluminum phthalocyanine (AlClPc), and lead phthalocyanine (PbPc).^93^

Polymorphism refers to the ability of molecules to form multiple distinct crystal structures. Controlling polymorphism in organic thin-films is challenging since π-conjugated molecules typically have similar cohesive energies and a low kinetic barrier to solid–solid transformations, making polymorphs difficult to isolate and stabilize.^88^ Common methods of obtaining different polymorphs in thin-films is through varying film thickness, temperature, surface chemistry and post deposition processes such as thermal and solvent annealing.^88^ The identification of polymorphs and the differences in morphological, structural, and spectroscopic properties have been documented through electrical conductivity measurements,^94,95^ optical absorption spectra,^96–99^ electron paramagnetic resonance spectroscopy (EPR),^94^ XRD,^96,97,100^ nuclear magnetic resonance (NMR),^91^ Raman spectroscopy,^91,97,98,101^ Fourier-transform infrared spectroscopy (FTIR),^96,97^ and surface imaging.^96,97,99^

**Fig. 11:**
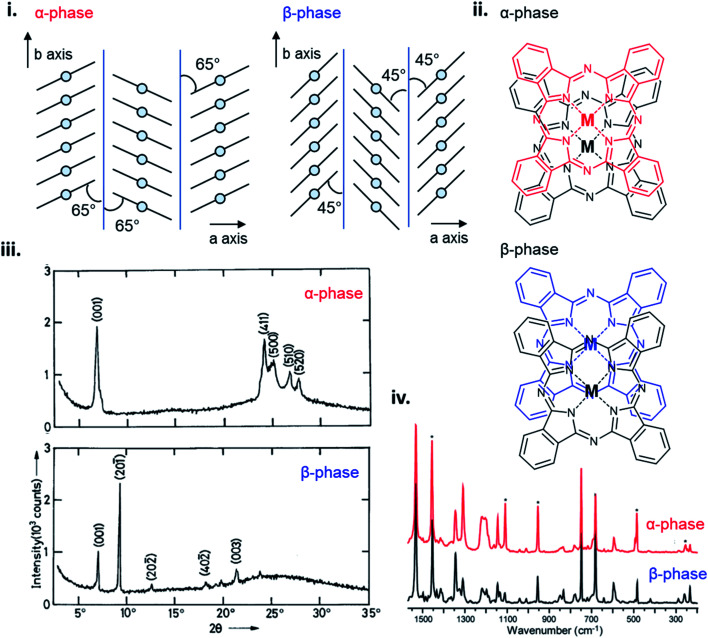
(i) Crystal packing structure of α-phase and β-phase CuPc. Reproduced with permission from ref. 96. Copyright© 2017 Elsevier B. V. (ii) α-Phase and β-phase superposition of phthalocyanine molecules along the *b* axis. Reproduced with permission from ref. 112. Copyright© 1988 American Chemical Society. (iii) XRD trace of α-phase and β-phase CuPc. Adapted with permission from ref. 100. Copyright© 1992 Wiley-VCH Verlag GmbH & Co. KGaA. (iv) Raman spectra of α-phase (red) and β-phase (black) CuPc. Adapted with permission from ref. 91. Copyright© 2010 American Chemical Society.

The polymorphic character of MPcs was first reported by Hamm and Norman in 1948 for CuPc^102^ and has since been extensively studied in a number of MPcs.^33,101,103–109^ MPcs are known to exists in various polymorphic forms identified as α, β, γ, δ, ε, and x-phases with the metastable α-phase and stable β-phase being the most common and commercially significant.^94,100,101^ The phase transition from α to β occurs in most MPc thin-films through exposure to temperature (200–300 °C)^97,99,100,104^ or organic solvents,^110,111^ and is characterized by a change in tilt angle between planes and the degree of π-electron overlap (Fig. 11i and ii).^96,112^ The stable β-phase is monoclinic in structure and forms long crystallite needles,^113^ whereas the metastable α-phase has been reported to be tetragonal,^114^ orthorhombic,^115^ or monoclinic^94^ in structure, and generally forms into spherical crystallites. As an example, Fig. 11 highlights some of the differences between α- and β-phase CuPc polymorphs. For both polymorphs the CuPc molecules align in the herringbone packing structure with a 65° angle between molecules and the *b* axis for α-phase CuPc and a 45° angle for β-phase CuPc.^96^ The larger angle of α-phase CuPc results in increased π-electron overlap and is likely the reason for the higher conductivity displayed by this polymorph.^94,95^ The XRD pattern of α- and β-phase CuPc (Fig. 11iii) shows the distinct crystallographic differences between polymorphs. α-Phase CuPc exhibits a primarily polycrystalline structure with crystallites preferentially oriented with their (001) planes (approximately 2*θ* = 7°) parallel to the substrate.^97,98,100^ In the case of β-phase CuPc alignment in the (20−1) direction is preferred as seen by the high intensity peak at approximately 2*θ* = 9°.^97,98,100^ Through Raman spectroscopy differences in the vibrational frequencies of α- and β-phase CuPc are shown in Fig. 11iv. Vibrational shifts between polymorphs can be observed in five Raman bands with the largest differences exhibited by the *ν*_52_ vibration (Cu–N deformation), *ν*_14_ vibration (C–H bending of the benzene ring), and the *ν*_28_ vibration (stretching of the phthalocyanine macrocycle).^91^ Differences in CuPc packing structure determine solid state properties such as conductivity, optical absorbance, and even colour which are critical for determining appropriate use in some applications. α- and β-phase CuPc are often used as organic semiconductors in electronic devices with particular interest in α-phase CuPc due to the high carrier mobility and high-frequency capacitance and conductance demonstrated by α-phase CuPc OTFTs,^116^ and α-phase CuPc–Si hetero-structures.^117^ Additionally, in the ink industry the most widely used blue pigments are CuPc based, with α- (purple), β- (green-blue), and ε-phase (red) CuPc being the most popular in printing inks, paints, plastics, and textiles.^91,118^

### 4.2 Thin-film morphologies

**Fig. 12:**
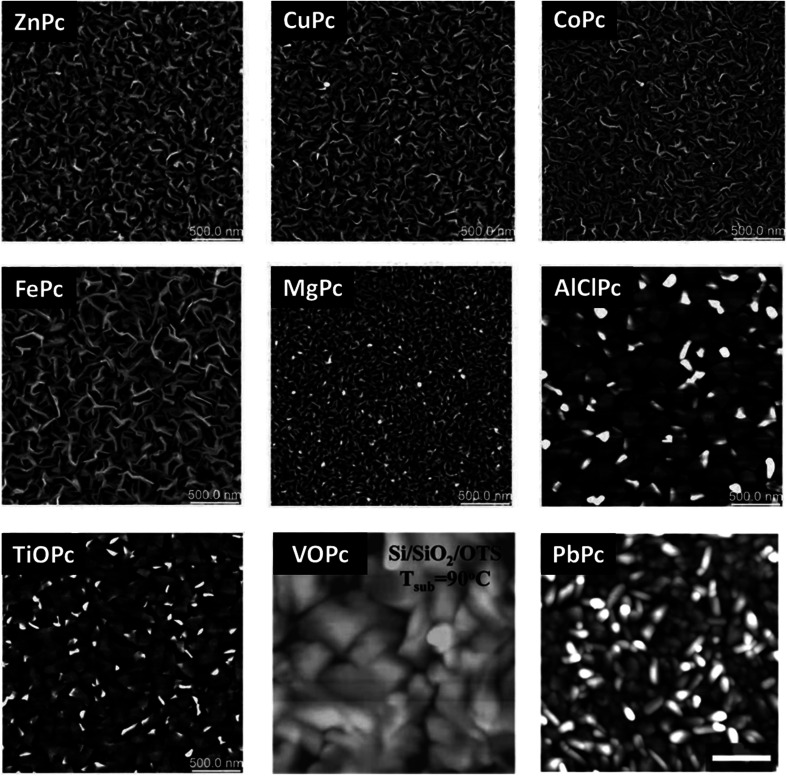
AFM images (2.5 μm × 2.5 μm) of CoPc, AlClPc, FePc, MgPc, TiOPc, ZnPc, CuPc deposited at *T*_s_ = 140 °C. Adapted with permission from ref. 119. Copyright© 2019 The Royal Society of Chemistry. AFM images (1 μm × 1 μm) of VOPc deposited at *T*_s_ = 90 °C. Adapted with permission from ref. 122. Copyright© 2008 American Chemical Society. AFM image (2 μm × 2 μm) of PbPc deposited at *T*_s_ = 70 °C. Adapted with permission from ref. 123. Copyright© 2011 American Chemical Society.

MPcs will form different surface morphologies depending on the molecular structure and corresponding packing. Fig. 12 displays AFM images of a number of MPc thin-films deposited by PVD onto heated substrates, including planar and non-planar structures and divalent, trivalent, and tetravalent metal inclusions. The planar divalent MPcs, such as ZnPc, CuPc, cobalt phthalocyanine (CoPc), iron phthalocyanine (FePc), and magnesium phthalocyanine (MgPc) exhibit comparable morphologies with ribbon-like grains of similar structure and shape with only small variations in grain size.^119^ Typically, ribbon-like grain morphologies are observed for films deposited on heated substrates whereas smaller more cylindrical shapes are observed at lower substrate surface temperatures.^32,33,120,121^ The non-planar trivalent and tetravalent MPcs, such as AlClPc, TiOPc, PbPc, and vanadyl phthalocyanine (VOPc) have much larger rectangular plate-like features owing to their different π-stacked packing structure.^119,122–126^ Additionally, these four MPc thin-films have a greater surface roughness and lower surface area to volume ratio compared to the planar divalent MPc thin-films.^119,122–125^ Unlike metal center, fluorination of the outside ring (F_*x*_MPc, *x* = 4, 8, 16) yields little effect on the morphology of MPc thin-films as studied in perfluorinated CuPc and ZnPc.^9,127–129^ In general the addition of fluoro molecules to the outside ring slightly alters grain size however, the packing structure and grain shape remain analogous to non-fluorinated MPcs.^9,127–129^

**Fig. 13:**
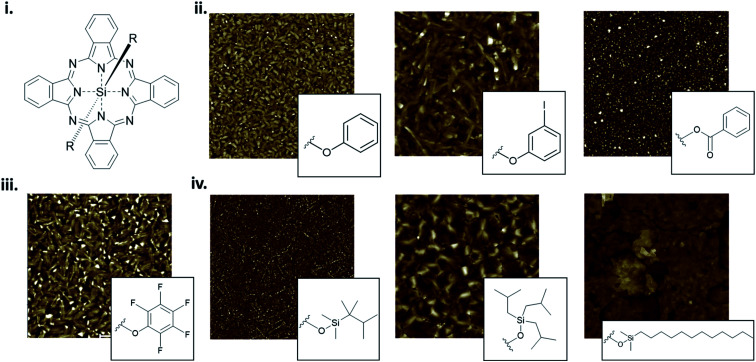
(i) Structure of axially substituted R_2_-SiPc. AFM images of R_2_-SiPcs with (ii) phenoxy, (iii) pentafluorophenoxy and (iv) alkyl axial substituents. Adapted with permission from (ii) ref. 130, (iii) ref. 131, and (iv) ref. 132. Copyright© 2020 American Chemical Society. Copyright© 2019 Wiley-VCH Verlag GmbH & Co. KGaA.

The packing and resulting thin-film morphology of MPcs can also be greatly altered through the inclusion of axial substituents as demonstrated by the AFM images of R_2_-SiPc presented in Fig. 13. R_2_-SiPc thin-films with phenoxy and fluorophenoxy groups fabricated by PVD (Fig. 13ii and iii) show two distinct morphologies either consisting of small regular circular grains or more elongated rectangular grains depending on the structure of the phenoxy substituent.^130,131^ Additionally, R_2_-SiPc molecules with alkyl axial substituents fabricated by solution processing (Fig. 13iv) highlight how the alkyl chain length, branching position and symmetry affect thin-film morphology; creating films with either small dense cylindrical grains, large interconnected grains, or very large plate-like features.^132^ By changing only the axial substituent, wide variations in thin-film morphologies are observed, where in general, it is hypothesized that large substituents alter molecular packing resulting in morphologies with sizeable features as demonstrated in R_2_-SiPcs and other conjugated small molecules.^132–134^ In electronic devices, morphology has been shown to impact the charge carrier mobility of transistors,^24,135,136^ the power conversion efficiency of solar cells,^137–139^ and the performance of light emitting diodes.^140^ Additionally, the mechanical stability of thin-films, including the flexibility and sensitivity to stress and strain, will affect the degree of reorganization in film morphology with mechanical deformation.^141–143^ In particular, films with large grains and broad boundaries are more susceptible to mechanical deformation as the formation of wide interconnected cracks are more prevalent compared to films with smaller grains and a smoother surface morphology.^141,143,144^

## 5. Physical properties of metal phthalocyanines

### 5.1 Absorption properties

**Fig. 14:**
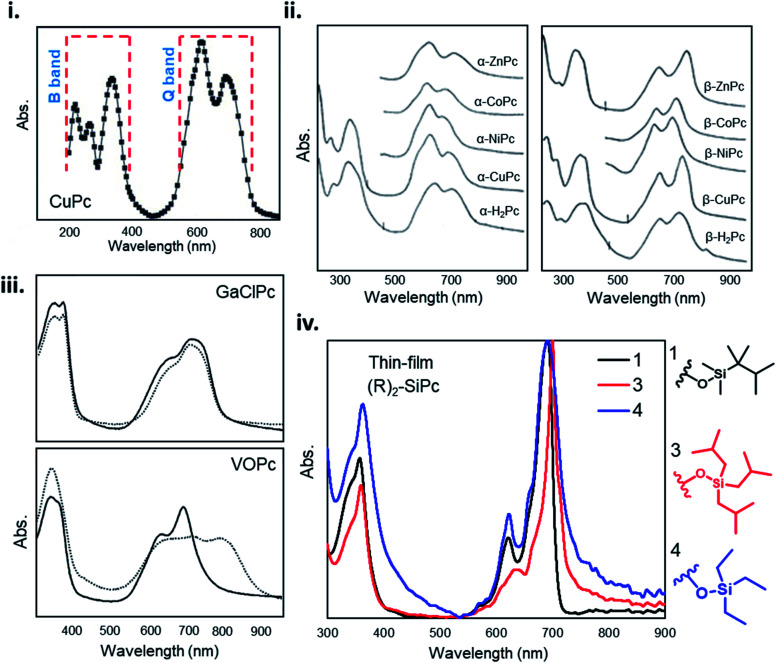
(i) Absorption spectra of as-deposited CuPc thin-film. Adapted with permission from ref. 147. Copyright© 2006 Elsevier Ltd. (ii) Absorption spectra and of α- and β-phthalocyanine thin-films. Adapted with permission from ref. 153. Copyright© 1968 American Institute of Physics. (iii) Absorption spectra GaClPc and VOPc thin-films as-deposited (straight line) and after the thermal annealing (dotted line). Adapted with permission from ref. 149. Copyright© 2004 Elsevier B. V. (iv) Absorption spectra of axially substituted R_2_-SiPcs thin-films. Adapted with permission from ref. 132. Copyright© 2020 American Chemical Society.

The unique ultraviolet-visible (UV-vis) absorption spectra of MPcs are a result of their extensively conjugated π-electron systems and the overlapping orbitals of the central metal. UV-vis spectroscopy is often performed on liquid samples which display sharp, well defined peaks. However, solvent coordination and aggregation can result in peak shifts uncharacteristic to the MPc itself.^145^ Additionally, solid state UV-vis absorption includes effects of thin-film molecular packing and crystal structures that are not visible in solution. MPcs typically display two strong absorption bands, one in the UV region of 280–350 nm known as the B (Soret) band, and the stronger, often better resolved, band in the visible region between 550–750 nm known as the Q band (Fig. 14i).^146–148^ For most planar MPcs, the B band displays three peaks and two shoulders as exhibited by CuPc in Fig. 14i,^147^ whereas non-planar MPcs display one to two broad peaks as seen in chloro-gallium phthalocyanine (GaClPc) and VOPc films (Fig. 14iii).^149,150^ In the low energy region of the B band (around 288 nm) changes to the absorption spectra between MPcs is thought to be due to orbital overlap of the phthalocyanine ring and the central metal.^146,147^ The high intensity peak in the low energy B band region exhibited in CuPc, CoPc, FePc, and nickel phthalocyanine (NiPc) suggests d-band association with the central metal, arising to π–d transitions as a result of the partially occupied d-orbitals of these metals.^146,147^ Changes in the higher energy region of the B band (210–275 nm) are thought to be a result of d–π* transitions.^146,147^

For all MPcs, the Q band region displays a single peak with characteristic Davydov splitting.^99,100,146,151–153^ In contrast, metal free phthalocyanine (H_2_Pc) can exhibit a split Q band due to asymmetry of the isoindole nitrogen atoms.^148,154^ The Q band has been interpreted in terms of π–π* excitation between bonding and anti-bonding molecular orbitals.^99,146,147,155^ The high energy peak of the Q-band has been assigned to the first π–π* transition on the MPc macrocycle with the low energy peak explained as either a second π–π* transition,^155^ an excitation peak,^156^ a vibrational internal interval,^157^ or a surface state.^157^ The extent of Davydov splitting observed in the Q band is related to the degree of available molecules able to participate in electronic transitions, in particular interactions between the transition dipole moments from adjacent molecules.^99^ Davydov splitting is common in all MPcs, however is more prominent in films which adopt a herringbone packing structure as seen by the spectra of CuPc and GaClPc in Fig. 14.^151,152^ This is also evident by Q band shifts and intensity changes of α- and β-MPcs UV spectra (Fig. 14ii), demonstrating how packing angle and therefore the degree of π-electron overlap alters Q band absorption.^153^

Several factors can influence Q and B band absorption, mainly the metal center and the inclusion of substituent groups. MPcs with different metal centers can lead to a Q band shift of around 100 nm as a function of metal size, coordination, and oxidation state. MPcs with closed-shell metals (lithium, magnesium, and zinc) typically exhibit a red shifted (bathochromic shift) maximum Q band peak, while open-shelled metals (iron, cobalt, and ruthenium) display a more blue shifted (hypsochromic shift) maximum peak due to stronger interactions with the phthalocyanine macrocycle.^148,151^ The chemical tunability of MPcs facilitates the functionalization in the peripheral, bay, and axial positions providing control over the physical, optical, and electronic properties. Functional groups can generally be categorized as electron withdrawing, such as sulfonyl, carboxyl, and fluoro groups, and electron donating, such as amino, alkoxy, and alkyl groups. Peripheral substituents with electron withdrawing character typically result in a red shifted Q band, whereas electron donating groups have little effect on the Q band absorption in solution samples.^148,151^ However, the addition of substituent groups may impact the molecular packing in thin-films and thus result in changes to the absorption spectra of solid samples. Additionally, functionalization at the bay position tends to result in a greater change in the absorption spectra of MPcs compared to similar groups in the peripheral position.^148,151^ The addition of axial substituents to MPcs will similarly affect the absorption spectra by altering the molecular packing resulting in shifted peaks of different intensity exhibited by the thin-film UV-vis spectra of axially substituted R_2_-SiPc shown in Fig. 14iv.^132,158^ The general trends relating MPc functionalization and absorption properties may not always hold true since the effects of added substituent groups will depend on the individual nature, number, and position of the group.

### 5.2 Vibrational properties

**Fig. 15:**
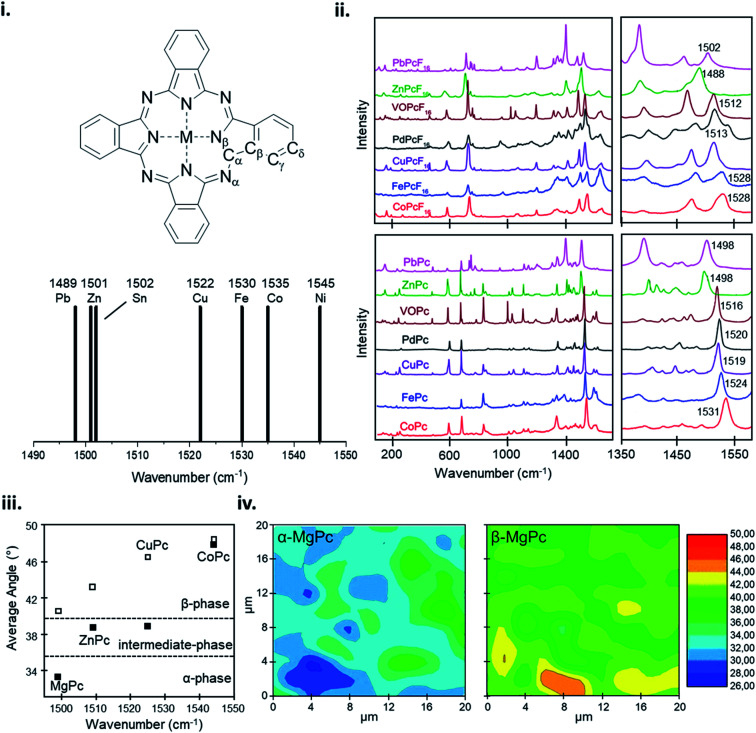
(i) Labelling scheme for MPcs and variation in position of the Raman band identified as an ion size marker. Adapted with permission from ref. 159. Copyright© 2001 PCCP Owner Societies. (ii) Raman spectra of MPcs and F_16_MPcs (M = Co, Fe, Cu, Pd, Zn, VO, Pb). Adapted with permission from ref. 160. Copyright© 2019 Elsevier B. V. (iii) Average angle between MPc molecule (M = Mg, Zn, Cu, Co) and substrate, and (iv) α-phase and β-phase angle maps between MgPc molecule and substrate estimated from polarized Raman spectra. Adapted with permission from ref. 101. Copyright© 2011 the Authors under the Creative Commons Attribution and Non-commercial License.

The vibrational properties of MPc thin-films can elucidate changes to the configuration of the MPc macrocycle as a result of substituent groups or large central metals, and insight into the orientation and packing structure of MPc molecules relative to the substrate. The vibrational modes of MPc thin-films assessed by both Raman and IR spectroscopy exhibit very similar spectra with the same structural trends and characteristic vibrational bands observed in powder samples and calculated data.^101,159–163^ Raman spectroscopy of MPc thin-films exhibit a distinctive band pattern with vibrations under 600 cm^−1^ attributed to the deformation of the macrocycle ring, N–M stretching, and the deformation of isoindoles.^164–166^ The 600–900 cm^−1^ vibrations are generally due to the deformation of the benzene and isoindole rings, with 1330–1445 cm^−1^ assigned to isoindole stretching and vibrations of the N–M and C–H bonds.^164–166^ The most intense vibrational band observed in MPcs is around 1500 cm^−1^ which exhibits a clear sensitivity to changes in the central metal with a definite trend correlating metal size to shifts in vibration.^159–161,163,164^ Bands in this region correspond to the displacement of the C_α_–N_β_–C_α_ bridges between isoindole groups in the MPc macrocycle (Fig. 15i).^159–161,163,164^ The change in wavenumber of this band observed in different MPcs correlates to the cavity size (N_α_–M–N_α_ distance) of the phthalocyanine macrocycle.^159,160^ MPc cavity size varies widely depending on the central metal with four possibilities: (i) the metal is smaller than the cavity size, (ii) the metal is approximately equal to the cavity size, (iii) the metal is larger than the cavity size, and (iv) the metal is much larger than the cavity size.^159^ These four scenarios result in either ring contraction, equilibrium ring geometry, ring expansion, or non-planar geometry and ring doming.^159^ Consequently, due to its high intensity this band allows for the identification of the central metal ion and orientation of MPc molecules in thin-films.^101,162,167^ Using the integral intensity obtained from polarized Raman spectra the angle between the MPc molecule and the substrate can be determined and used to ascertain the effects of fabrication parameters such as substrate temperature, deposition method, and film thickness, and identify polymorphic phases and film order.^101,162,167^ For MPcs with a cavity diameter similar to that of H_2_Pc (3.93 Å) such as CoPc, FePc, CuPc, and manganese phthalocyanine (MnPc) a planar equilibrium geometry is adopted.^159^ With a cavity diameter of 3.66 Å, NiPc is an example of an MPc with a metal inclusion that is smaller than the cavity of the phthalocyanine ring such that the four isoindole groups are pulled towards the metal center resulting in ring contraction.^159^ Conversely, ZnPc with a cavity diameter of 3.96 Å is an example where the metal is larger than the cavity of the phthalocyanine ring causing ring expansion but not large enough to result in a non-planar geometry.^159^ Lastly, PbPc and tin(ii) phthalocyanine (SnPc) have much larger metal centers and are pushed out of the MPc ring resulting in a non-planar geometry and ring doming.^159^ The effects of metal ion size on the MPc macrocycle are observed by shifts in the vibrational band corresponding to the C_α_–N_β_–C_α_ bridge bonds, with the wavelength noticeably decreasing with an increase in metal size.^159,160^ NiPc has the most shifted position at 1545 cm^−1^ (Fig. 15i), with all other MPcs ordered according to metal size (Fig. 15i and ii).^159^ Although ZnPc, PbPc, and VOPc have similar located bands in the lower wavenumber region, PbPc and VOPc display significant ring doming and a non-planar geometry, suggesting that the packing structure has less of an impact on the vibration properties compared to metal ion size.^159,160^ Additionally, this trend holds for fluorinated MPcs as seen in Fig. 15ii, with a more dramatic shift observed in F_16_MPcs compared to their non-fluorinated analogs as the addition of fluoro substituents has a noticeable impact on the N_α_–M–N_α_ distance.^160^ Other than the metal dependant band around 1500 cm^−1^, the spectra region from 1350–1500 cm^−1^, known as the finger print region, changes depending on the individual MPc and can display up to six unique bands.^159,160^ This region has been known to change depending on the metal center, degree of fluorination, and the inclusion of substituent groups.^159,160,164^

A change in metal ion band intensity in MPc films is attributed to changes in the molecular packing and film organization whereas band location is a result of metal ion size.^101,162^ Polarized Raman spectroscopy using parallel and cross polarization allows Raman surface mapping to determine the angle distribution of MPc molecules in thin-films and the identification of polymorphic forms (Fig. 15iii and iv). The change in MPc orientation can be observed by an increase or decrease in band intensity with different polarizations, indicating a change in angle between MPc molecules and the substrate. Szybowicz *et al.*, demonstrated this through the polymorphic forms of various MPcs studied by polarized thin-film Raman spectroscopy.^101,162,167^Fig. 15iii shows the average angle between MPc molecules and the substrate determined by the C_α_–N_β_–C_α_ bridge vibration before and after thermal annealing to induce a polymorphic phase transition.^101^ For the MPcs studied an increase in angle was observed between the substrate and MPc, with a smaller increase exhibited by MPcs with a large cavity diameter (ZnPc) compared to MPcs with a cavity size similar to that of H_2_Pc (CuPc and MgPc).^101^ The Raman surface map reveals additional information on the angle and orientation of MPc molecules in films. Using MgPc as an example Fig. 15iv shows the angle distribution of molecules estimated by polarized Raman surface mapping before and after thermal annealing.^101^ Before annealing, the film consists of the metastable α-phase with molecules aligned 26–36° to the substrate while after annealing Raman mapping shows the transition to the more stable β-phase with molecules aligned 39–46° to the substrate.^101^ Through Raman and IR spectroscopy the vibrational properties of MPc thin-films can be used to determine fundamental thin-film characteristics such as molecular alignment and film homogeneity, and identify MPc films by their metal ion and polymorphic forms.

## 6. Synchrotron techniques for thin-film characterization

**Fig. 16:**
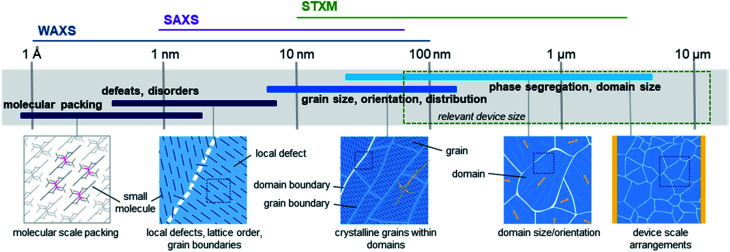
Size scales, structural features, and relevant X-ray characterization techniques for organic thin-films. Adapted with permission from ref. 168. Copyright© 2012 American Chemical Society.

High performing organic thin-film devices rely on the specific interfacial orientation and alignment of molecules to achieve optimum opto-electric properties and thus the characterization of these molecular interfaces is critical to the development of advanced devices. The variable nature of organic thin-films can lead to an imbalance in property optimization where often the ability to fine tune molecular structure to optimize nano-scale properties, such as intermolecular charge transfer, negatively impacts large-scale thin-film formation properties. From molecular packing to crystallite formation, analysis of the thin-films must be performed at various size scales in order to fully characterize the films. Fig. 16 illustrates the relevant size scales and corresponding structural characteristics important to organic thin-films and the synchrotron based X-ray techniques which can be used to provide information at each scale.^168^ X-ray techniques using synchrotron light sources provide additional information not possible with other methods like optical microscopy, scanning probe techniques, or transmission electron microscopy (TEM).^168^ The ability to select specific wavelengths and vary the incident and collection angles facilitates the resolution of nano-scale features such as bond lengths, molecular packing, and phase segregation through the entire film, rather than strictly at the surface. Additionally, unlike lab scale X-ray methods, synchrotron X-ray techniques can be used to study weakly scattering samples due to the greater flux, brilliance, and collimation of synchrotron light sources, making them ideal for investigating organic thin-films.^168^

**Fig. 17:**
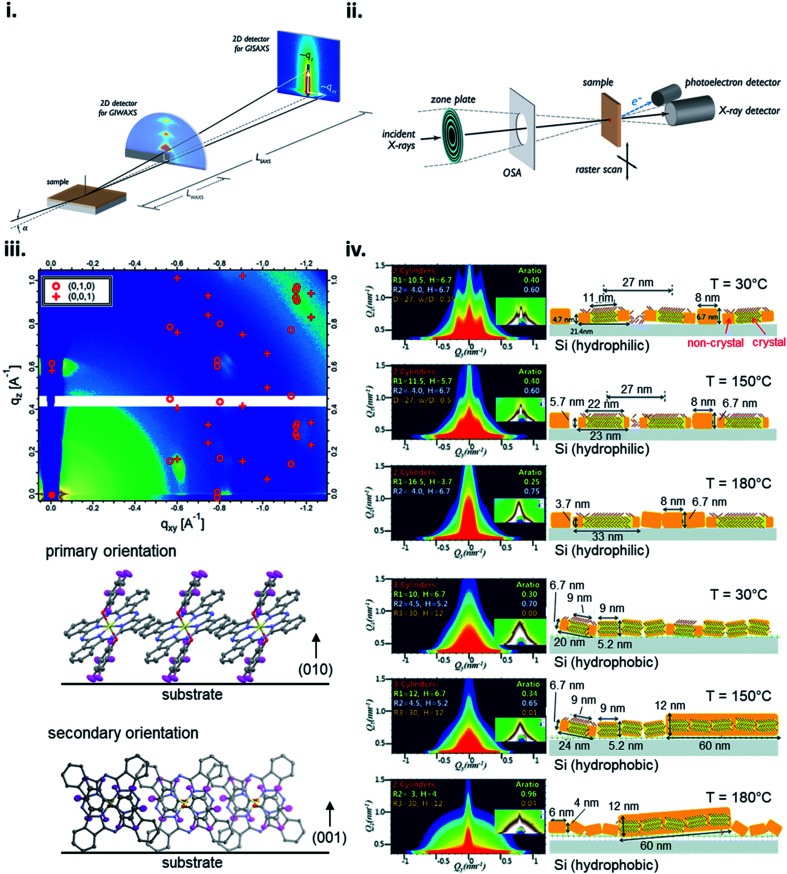
Schematic diagram of (i) GIXS, and (ii) STXM. Adapted with permission from ref. 168. Copyright© 2012 American Chemical Society. (iii) 2D GIWAXS pattern and schematic diagram of molecular orientation of bis(pentafluoro phenoxy) SiPc (F10-SiPc) thin-film. Adapted with permission from ref. 130. Copyright© 2020 American Chemical Society. (iv) 2D GISAXS patterns and schematic diagrams of molecular orientation showing the thermal evolution of CuPc thin-films on hydrophilic and hydrophobic Si surfaces. Adapted with permission from ref. 169. Copyright© 2012 The Royal Society of Chemistry.

X-ray scattering techniques employ the distribution of incident X-rays through a sample where a fraction of the waves are diffracted and collected creating distinct diffraction patterns with high intensity peaks characteristic to the specific film properties. The angle of the diffracted peaks provides information on the spacing between molecular planes in the film, whereas the direction of the peaks correspond to the orientation of the planes. Grazing-incidence X-ray scattering (GIXS) is a common X-ray scattering technique where the scattering vector is directed along the sample plane and the diffracting planes are perpendicular to the sample plane.^168^ GIXS can be used to analyze the bulk or surface film properties depending on the chosen incident angle and detection method, for example signal can be collected by a point detector for high accuracy or more commonly using a 2D detector for rapid data collection over a large area with minimal sample damage (Fig. 17i).^168^ Grazing-incidence wide-angle X-ray scattering (GIWAXS) and grazing-incidence small-angle X-ray scattering (GISAXS) are two of the most commonly used synchrotron techniques to investigate organic thin-films with the ability to resolve features in the range of approximately 1 Å – 100 nm for GIWAXS and 1–100 nm for GISAXS.^168^ 2D GIWAXS patterns can be used to determine crystal packing through the size and symmetry of the unit cell by analysing peak position and intensity, crystallite size and disorder by analysing peak width, and the degree of crystallinity by analysing the integrated diffraction intensity.^168^ Additionally, the molecular orientation and alignment can be determined by performing an azimuthal scan where a diffraction peak is selected and the intensity recorded while the sample is rotated about the substrate normal to determine the orientation distribution.^168^ GIWAXS has been demonstrated to be useful for determining how fabrication parameters, such as annealing temperature, effect the molecular orientation of small molecules,^56,73,132^ and how molecular structure effects orientation as demonstrated by R_2_-SiPc thin-films (Fig. 17iii).^130,132^ GISAXS is used to analyze the nano-scale surface morphology of polymer and multi-component thin-films with some use in quantifying domain size in single-component small molecule films as demonstrated in Fig. 17iv which characterizes CuPc thin-film formation using different annealing temperatures on different surfaces.^168–170^ However, GISAXS is predominantly used to study the phase segregation and morphology in polymer and small molecule-polymer blends and is typically used in conjugation with GIWAXS in order to obtain a more complete analysis.^168,169^

Scanning X-ray microscopy techniques combine X-ray scattering or spectroscopy methods with a spatially resolved rendering of an image using rasters through a focused X-ray beam (Fig. 17ii).^168,171^ Scanning transmission X-ray microscopy (STXM), often called near-edge X-ray absorption fine structure spectroscopy (NEXAFS) microscopy, is a common method which combines high resolution images with NEXAFS spectra to obtain composition and orientation maps of single and multi-component thin-films.^168,171^ Typically STXM is used for large-scale features (10 nm to 5 μm) and similar to GISAXS has found the most utility in films consisting of polymer and small molecule-polymer blends.^171–173^ Orientation and order mapping of single-component thin-films is achieved by polarized STXM measurements. Different molecular orientations with respect to the polarization axis can be determined by tuning the photon energy to a specific dichroic NEXAFS resonance while measurements with a linearly or elliptically polarized X-ray beam provide contrast between molecules.^168,171^ Thus, by rotating the sample about the surface normal and collecting multiple images in the same region with different in-plane polarizations, areas of varying molecular orientation can be mapped and information such as packing structure, tilt angle, and domain size can be acquired for the bulk film.^168,171^ Therefore making STXM a useful tool for large area visualization of organic thin-films with recent use demonstrated in analyzing the composition of bis(tri-*n*-propylsilyl oxide) SiPc/poly-(3-hexithiophene) blends in thin-films.^173^

## 7. Conclusion

For over 90 years MPcs have demonstrated their utility as colourants, catalysts, and semiconductors, with particular interest as thin-film active layers in a myriad of electrical devices. With nearly endless molecular structure possibilities, the ongoing research into the physical, chemical, mechanical, electrical, and optical properties of MPc thin-films is an evolving discipline. Understanding the building blocks in the formation of MPc thin-films from deposition, to nucleation and film growth, helps recognize the influences of chemical structure and fabrication conditions on film microstructure, morphology, and properties. Herein the fundamentals of small molecule nucleation and growth in the context of MPc thin-films fabrication by PVD and solution processing have been discussed with focus on the thermodynamic and kinetic considerations, and how various fabrication parameters and methods effect film formation. The structure-property relationship of MPc thin-films was considered in terms on film microstructure, surface morphology, and optical and vibrational absorption properties. This review provides a valuable resource for the design and application of MPc based thin-films.

## Conflicts of interest

There are no conflicts to declare.

## Supplementary Material
